# Dissecting the schistosome cloak

**DOI:** 10.7554/eLife.36813

**Published:** 2018-04-27

**Authors:** Carolyn E Adler

**Affiliations:** 1 College of Veterinary MedicineCornell UniversityIthacaUnited States

**Keywords:** Schistosoma mansoni, Tegument, Stem Cell, Tropical disease, Schistosomiasis, Zinc-finger protein, Other

## Abstract

Two proteins required for the growth of a skin-like structure called the tegument in parasitic flatworms could be new targets for drugs to kill these parasites.

**Related research article** Wendt GR, Collins JN, Pei J, Pearson MS, Bennett HM, Loukas A, Berriman M, Grishin NV, Collins JJ. 2018. Flatworm-specific transcriptional regulators promote the specification of tegumental progenitors in *Schistosoma mansoni*. *eLife* **7**:e33221. doi: 10.7554/eLife.33221

Schistosomiasis is a devastating disease that affects around 200 million people worldwide. It is caused by parasitic flatworms known as flukes or schistosomes, which can infect people through exposure to contaminated drinking water and poor sanitation. Current treatments only work after infection has occurred, which makes it difficult to eradicate this disease completely (World Health Organization, 2018).

Schistosomes belong to the animal clade called neodermata, which consists of approximately 100,000 species of parasitic flatworms (Littlewood, 2006). The key evolutionary innovation defining this clade is a skin-like structure called the tegument that allows the parasites to withstand particularly harsh environments, such as the human digestive system and the bloodstream. It also helps the worms to absorb nutrients and attach to their hosts, important adaptions that potentially enabled the worms to become parasites.

Since all parasitic flatworms have a tegument, it is a prime target for drug development. Indeed, the only drug that is currently available for the treatment of schistosomiasis, praziquantel, is thought to work by dissolving the tegument, although the mechanisms involved remain unknown (Chai, 2013). We also do not fully understand how adult schistosomes generate and maintain their teguments.

Some studies indicate that schistosomes can survive inside human hosts for decades without getting detected by the immune system (Basch, 1991). Until recently, visualizing the tegument has required the use of electron microscopy, a technique that is difficult to combine with other strategies for highlighting cells. Now, in eLife, James Collins and colleagues at the University of Texas Southwestern Medical Center (UTSW), James Cook University and the Wellcome Trust Sanger Institute – including George Wendt of UTSW as first author – report a straightforward method to label the tegument with fluorescent dyes (Wendt et al., 2018).

Soaking mature schistosomes in water causes the tegument to swell, which damages its membrane and so allows the fluorescent dyes to seep into it. Unlike electron microscopy, fluorescent dyes are compatible with other cell biology techniques, such as lineage-tracing, in situ hybridization and antibody labeling. By using these methods, Wendt et al. were able to determine the identity and birthdate of the cells in the tegument. The results showed that far from being a static structure, the tegument constantly incorporates new cells, which are produced by adult stem cells (Figure 1; Collins et al., 2013). The rapid turnover of cells in the tegument resembles the production of epithelial cells in free-living flatworms called planarians, from which parasitic flatworms have evolved (Laumer et al., 2015; Egger et al., 2015).

**Figure 1.**
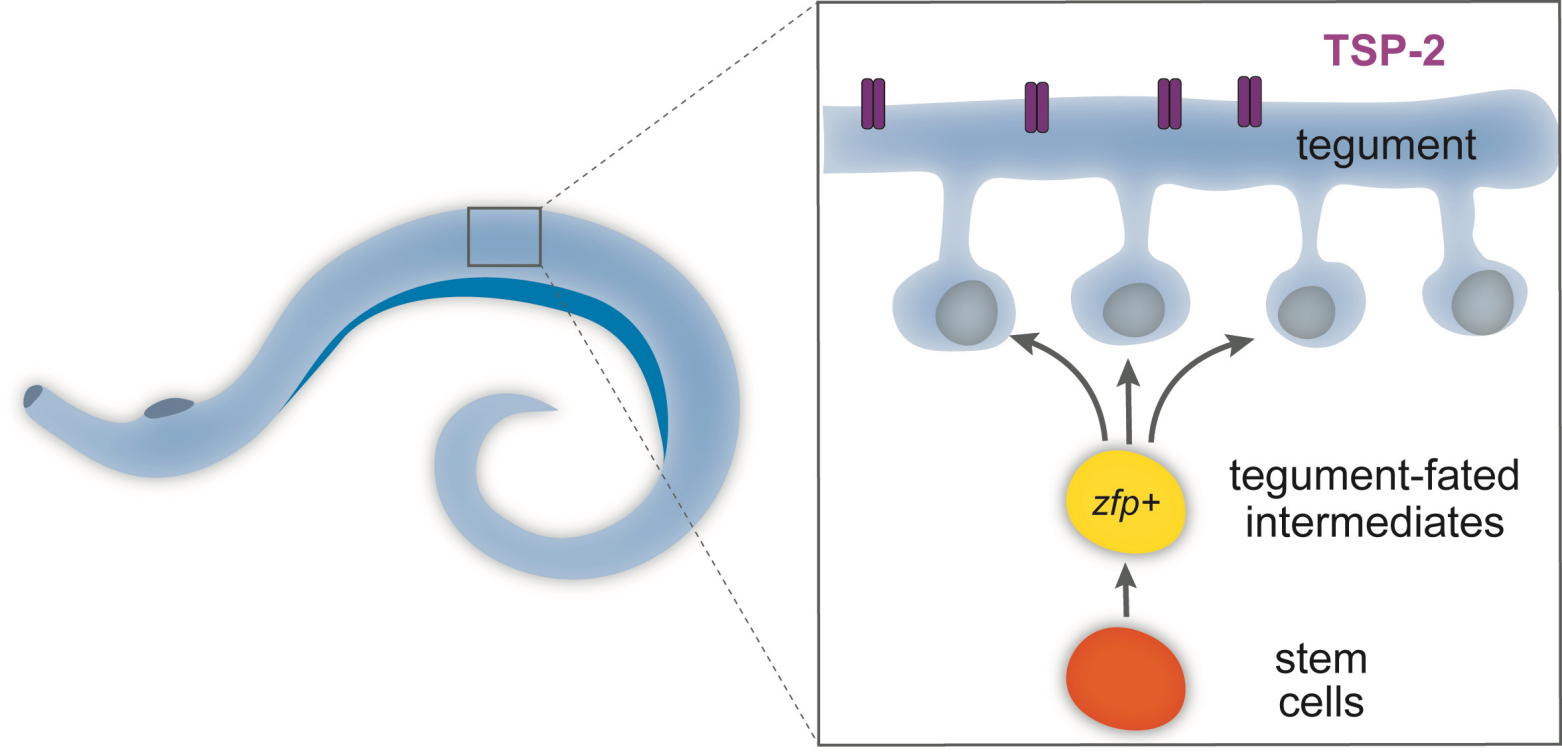
Schematic of the tegument in adult flatworms. Parasitic flatworms (schistosomes) live in the human bloodstream and are covered by a skin-like structure called the tegument, which is constantly replenished by newly produced stem cells (see inset) and so protects the worms from being detected by the host immune system. The tegument (blue) is made of cells that have fused together to form a continuous structure and express a protein called TSP-2 (purple). The cells in the tegument derive from stem cells (orange). These stem cells divide to produce intermediate cells (yellow), which mature to produce the cells (blue) that fuse to form the tegument. These tegument-fated intermediate cells express ‘zinc-finger’ proteins (zfp) and are necessary to build the skin-like structure. Creating drugs that could block these genes may present a new opportunity to treat schistosomiasis.

Wendt et al. then used an antibody that recognizes TSP-2: this protein, which is found on the surface of the tegument, is a molecular target in efforts to develop a schistosome vaccine (Pearson et al., 2012). This antibody allowed the researchers to purify the tegument cells, to determine the genes that are expressed by these cells, and to identify the proteins that are required to build the tegument. Wendt et al. identified two ‘zinc-finger’ proteins, which are only found in flatworms. These two proteins are necessary for forming the cells that produce the tegument. When one of these proteins is blocked, the schistosomes can no longer adhere to substrates, which may affect their ability to interact with the host.

Intriguingly, free-living planarians also rely on stem cells that express a similar zinc-finger protein to generate their epidermis (van Wolfswinkel et al., 2014; Tu et al., 2015). So, even though they have very different surfaces, parasitic flatworms and free-living flatworms employ strikingly similar molecular processes to make their ‘skin’. These conserved mechanisms may therefore be a prime target for drugs that treat schistosomiasis by preventing the formation of the tegument.

The dynamic nature of the tegument demonstrated in the study of Wendt et al. raises key questions about schistosomes and their host-parasite relationships. In particular, it is unclear how the integrity of the tegument can be maintained while its cells are constantly turning over. Over the course of its life, a schistosome displays antigens on its exterior that, presumably, reveal to its host that it is a foreign invader. So how does the worm regulate which proteins remain on its surface when new cells constantly join the tegument?

It has previously been hypothesized that rapid shedding of the tegument could be a mechanism to evade the immune system of the host, to potentially prevent antigens from being exposed (Abath and Werkhauser, 1996). More research is needed to determine how the constant replacement of the tegument cells contributes to the schistosome’s ability to evade host immune systems. Furthermore, expanding the repertoire of potential treatments remains a high priority, given the extreme number and diversity of parasitic flatworms, and the debilitating effects of the diseases they cause in livestock and in humans.
